# Perceived rehabilitation needs of older people with dementia: A qualitative interview study

**DOI:** 10.1186/s12877-025-06570-9

**Published:** 2025-11-07

**Authors:** Outi Mäenpää, Johanna Edgren, Mari Aaltonen, Lina van Aerschot, Jari Pirhonen, Jenni Kulmala

**Affiliations:** 1 Faculty of Social Sciences (Health Sciences) and Gerontology Research Center, Tampere University, Tampere, Finland; 2 Department of Healthcare and Social Welfare, Finnish Institute for Health and Welfare, Helsinki, Finland

**Keywords:** Dementia, Rehabilitation, Needs, Home care, Qualitative research

## Abstract

**Background:**

Rehabilitation is an umbrella term for actions that aim to optimise autonomy, life management, functioning, and well-being of older people. Physical rehabilitation supports physical functioning and daily activities. However, in dementia care, rehabilitation is an underutilised resource, and healthcare professionals describe challenges in implementing rehabilitation. Also, scientific knowledge of individual rehabilitation needs is relatively scarce. The aim of this qualitative study was to understand the perceived rehabilitation needs of older people with dementia.

**Methods:**

Participants were recruited from a care organisation that provides home- and long-term care and adult day care to community-dwelling older people. In total, 12 persons with the mean age of 83 years with mild to moderate dementia participated in this study. Data were collected through face-to-face focus group interviews and analysed using inductive thematic analysis.

**Results:**

The study participants described their rehabilitation needs through three main themes: (1) Need for coordination of rehabilitation, (2) Need for individually tailored rehabilitation, and (3) Need for social support. Rehabilitation was mainly seen as physical activity and physical rehabilitation. Participants felt that rehabilitation could take place both through self-directed activities, such as going for a walk, or participating in rehabilitative activities like group exercises, as well as through planned activities conducted with a rehabilitation professional or home care staff.

**Conclusions:**

This study identified several key elements for successful rehabilitation described by older people with dementia. The results highlight the importance of well-coordinated, individually tailored rehabilitation, and social support with distributed agency. These results can help healthcare professionals and family members to implement rehabilitation successfully for this rapidly growing special target group.

**Supplementary Information:**

The online version contains supplementary material available at 10.1186/s12877-025-06570-9.

## Background

Many people with dementia can maintain the ability to perform daily activities [1] and live in their own homes [2]. However, over time, dementia progressively impairs a person’s physical and cognitive abilities [3]. Persons with dementia often have other co-morbidities, which may accelerate functional decline [4, 5]. Cognitive decline, challenges in coping with activities of daily living (ADLs), and ADL dependency lead to increased care needs [6, 7] and potentially institutionalisation [8]. Rehabilitation may slow the progression of physical and cognitive decline in people with dementia [9–11], but they may not be able to request rehabilitation the same way as their cognitively healthy peers. To promote rehabilitation of people with dementia, we need to know more about how they identify and describe their rehabilitation needs.

To ensure that people with dementia and their carers can live well, and to reduce the impact of dementia on individuals and societies, the World Health Organization highlights the importance of promotive and preventive services, including rehabilitation, in their Global Action Plan on the Public Health Response to Dementia 2017–2025 [12]. Accordingly, a rehabilitative approach should be a key component of dementia care [12–14]; it is essential to daily care but is also a broader underlying care philosophy [13, 14].

Rehabilitation can be seen as an overarching concept for actions that aim to optimise autonomy, life management, functioning, and well-being of a person with dementia [15, 16]. These actions typically involve interdisciplinary and versatile approaches [15, 17, 18]. There are various forms of rehabilitation that have shown benefits for people living with dementia, such as cognitive rehabilitation, speech-and language therapy, occupational therapy and physical rehabilitation [10, 19–21]. This study focuses on physical rehabilitation, which aims to support physical functioning [22, 23] and can include, for example, therapeutic exercises, self-directed physical activity or training daily activities with the support of professionals such as physical therapists or occupational therapists [18, 22].

A person-centred approach has been proposed as a foundation of rehabilitation, supplemented with goal-setting and empowering principles [24]. Rehabilitation should be rooted in identified individual needs, strengths and goals [13, 25, 26]. According to the review by Jogie et al. (2021), the most common rehabilitation goals among people with dementia are related to improving functional impairments, activity and participation, often involving a combination of these areas [27]. In the study by Dutzi et al. (2019) people with dementia described maintaining mobility and supporting psychological well-being as the most important rehabilitation goals [26]. Overall, previous studies suggest that goal setting is feasible for people with dementia and collaborative goal setting can support participation and engagement in rehabilitation [26, 27].

The rehabilitation of people with dementia has many benefits in everyday life. Physical rehabilitation, in particular, has shown the potential to slow the progression of physical and cognitive decline of people with dementia [9–11]. Previous intervention studies have shown that physical rehabilitation not only slows the decline in physical and cognitive functioning but also supports the ability to perform daily activities [1, 10], reduces falls [9, 28], and alleviates behaviour changes [29]. Multidimensional interdisciplinary rehabilitation, including physical rehabilitation, may have the potential to reduce the risk of institutionalisation for home-dwelling people with dementia [30]. By slowing the deterioration of physical functioning and daily activities, it is possible to support their independence and safer daily life [9, 31, 32]. In addition, physical functioning and independence in ADLs are linked to the experience of subjective well-being [33] alongside psychosocial factors [34].

Despite the known benefits and WHO global goals, such as achieving physical, mental and social wellbeing for people with dementia [12], rehabilitation appears to still be an underutilised resource in dementia care [35, 36], and there seem to be inequities in accessing rehabilitation services [25, 37, 38]. In addition, healthcare and rehabilitation professionals may consider that implementing rehabilitation for people with dementia is challenging [39, 40] or that these individuals will not even benefit from rehabilitation [36, 40, 41]. People with dementia may also have specific needs, including a focus on therapeutic relationships, intricacies of movement and motor learning [42]. They also report challenges in engaging with rehabilitation [25] and reaching out for rehabilitative services [25, 43]. There are barriers to rehabilitation access both from the user and health service points of view, including a lack of knowledge about rehabilitation among both persons with dementia and healthcare professionals and non-existing or fragmented services that are difficult to navigate [43]. However, when rehabilitation is provided, people with dementia describe it as desirable and beneficial [25] and that it can lead to a sense of empowerment [44].

To provide applicable rehabilitation for older people, including those with dementia and their specific needs, it is essential to place their experiences and necessities at the center of planning, developing, and implementing rehabilitation services [12, 45]. However, scientific knowledge of rehabilitation needs described by people with dementia themselves is still relatively scarce. Understanding their perceived rehabilitation needs is essential for addressing the challenges related to rehabilitation, developing appropriate services, and thereby facilitating successful rehabilitation. Therefore, the aim of this qualitative study was to understand the perceived rehabilitation needs of older people with dementia.

## Methods

### Study design and participants

Phenomenology was used as a foundational philosophy in approaching the data to gain a deeper understanding of rehabilitation needs as experienced by persons living with dementia [46]. In this study, rehabilitation needs are seen as an individual’s lived experience, and instead of merely listing experienced needs, the study aims to capture the deeper meanings of these experiences.

Participants for this study were recruited from a care facility that had consented to participate; the facility provides home- and long-term care and adult day care to community-dwelling older people in the Pirkanmaa region in Finland. The organisation named a service coordinator to assist with the sampling. The service coordinator informed their clients about the study as neutrally as possible and encouraged potential participants to take part without pressuring them. The inclusion criteria were that the participants should (1) receive home care from the care facility, (2) have a cognitive decline, (3) be able to participate in group discussion, and (4) be able give informed written consent to participate in the study. With these criteria, 12 care clients agreed to participate; there were 10 women and 2 men, ranging in age from 73 to 93 years, the mean age being 84 years. The severity of dementia among participants varied from mild to moderate according to healthcare professionals who helped with the recruitment. Formal diagnoses or severity assessments were not verified by the research team, as access to participants’ medical records was not available.

### Data collection and analysis

Data were collected through face-to-face focus group interviews between March and June 2024. Interviews were conducted by two researchers. The first author (OM) was the lead interviewer, and the senior author (JP) observed and assisted, for example, by raising follow-up questions. The senior author had prior experience in designing and conducting focus group interviews with older adults with cognitive decline living at home and in care settings. Altogether, three focus groups, including three or four participants, were formed, and each group had two separate interview sessions, i.e., six interview sessions in total. All participants, except one, participated in both interview sessions. The interview sessions were 57–99 min in duration, the average being 74 min.

A semi-structured thematic interview guide was used as a framework for the interview. The first interview question: *‘What thoughts does the word “Rehabilitation” bring to your mind?’* was an opening question and it provided the researcher with an initial understanding of how participants living with dementia conceptualised rehabilitation and what assumptions they held, shaped by their personal experiences and cognitive context. The interview guide (Additional file 1) included open-ended questions such as: *‘Please describe your own wishes related to rehabilitation.”* Researchers encouraged the participants to share their experiences freely, and open-ended questions gave the participants an opportunity to elaborate on their experiences. The interviewers ensured that all participants could speak as equally as possible by facilitating turn-taking. In the interview sessions, the discussion progressed according to each group’s preferences. However, the interviewers ensured that all themes outlined in the interview guide were covered in each interview session. Already with the first focus group, it became evident that, considering the participants’ endurance and a reasonable time frame, it was necessary to divide the interview into two separate sessions. The interviews were audio-recorded by the interviewers and transcribed verbatim by a professional transcriber who was not involved in the study. To ensure confidentiality, all participants were pseudonymized, and any identifiable information was removed during the data processing. There were a total of 116 pages of transcribed interview data.

The first author analysed the data using inductive thematic analysis by following the process introduced by Braun and Clarke (2006) [47]. The first author, who had the main responsibility for data analysis, documented her pre-understanding (pre-conceptions, thoughts, knowledge and experiences) prior to data collection [48]. Aware of how her professional background in rehabilitation could shape the findings, the researcher actively sought to remain open to the data so that the participants’ experiences could be genuinely represented. Analysis progressed cyclically rather than strictly following a linear step-by-step pattern [47]. In the first phase of the analysis, data were reviewed multiple times, and preliminary notes were made. After gaining a comprehensive familiarity with the data, the original expressions that were relevant to the research question were marked, and the expressions generated into initial codes [47], which were organised into groups by identifying the meanings and relationships between the codes. These groups formed sub-themes, which were then developed into main themes. Throughout the analytical process, themes were flexibly constructed in reflective interaction between the researcher and the data [47, 48]. The first author discussed and agreed on the preliminary themes with co-authors. Moreover, Atlas.ti software was used to organise the data.

## Results

In general, the participants largely perceived rehabilitation as physical activity and physical rehabilitation. They described how rehabilitation could take place both through self-directed activities such as going for a walk or participating in rehabilitative activities such as group exercises, as well as through planned activities carried out with a rehabilitation professional or care workers.

We identified three main themes by which the participants described their rehabilitation needs (Table 1): (1) need for coordination of rehabilitation, (2) need for individually tailored rehabilitation, and (3) need for social support.

**Table 1:** Main and sub-themes describing the rehabilitation needs of older people with dementia

Main themes	Sub-themes
Main theme 1. Need for coordination of rehabilitation	1. Reliable identification of rehabilitation needs and systematic referral to rehabilitation 2. Accessible and low-threshold rehabilitation services
Main theme 2. Need for individually tailored rehabilitation	1. Genuine encounter and consideration of an individual’s characteristics 2. Adaptable and meaningful rehabilitation content
Main theme 3. Need for social support	1. Improving motivation and commitment in rehabilitation 2. Support for mental well-being

### Theme 1: need for coordination of rehabilitation

Based on the study participants’ experiences, identifying and accessing rehabilitation services seem to appear as fragmented. Their experiences suggest that there are shortcomings in referring individuals to rehabilitation, even in cases where the need is evident – such as after repeated falls. The participants seem to experience difficulties having their rehabilitation needs recognised by care staff, which leads to difficulties in accessing rehabilitation services and to a sense that rehabilitation is selectively available and not offered systematically. There seems to be a need for consistent and equal referral to rehabilitation, which requires systematic coordination.

*Yes*,* it’s quite a tough game*,* because in the end*,* people are selected for follow-up care in a harsh way. Prayer or anything else doesn’t help much with that. Because it’s been decided*,* and the people assigned to handle it will take care of it in their own way.* (Kate, woman 86 years)

However, while there appear to be shortcomings in how professionals facilitate access to rehabilitation, the study participants also acknowledged their own role and responsibility in seeking rehabilitation. The participants’ experiences reflect that several factors, such as a lack of personal initiative, may hinder access to or participation in rehabilitation. Based on the study participants’ experiences, accessing rehabilitation services requires proactive and coordinated support shaped by both professional guidance and individual initiative.

*I have not participated in rehabilitation*,* nor have I been asked or rehabilitated. But perhaps I just have not known how to request rehabilitation.* (Simone, woman 92 years)

*There is free rehabilitation and there is paid rehabilitation*,* but one thing that applies to me is that natural laziness plays a significant role*,* which comes from not bothering. I’m just so lazy. That is my own downside in this.* (Maria, woman 86 years)

In addition, as the following quotation reflects, there is thus a need for rehabilitation that is sustained and continuous in nature and should not be terminated prematurely.

*I had a physical therapist coming to see me when I first moved into this new apartment*,* which was four months ago. She came to get to know me and provided some preliminary guidance. But that was it; nothing else has been offered since then.* (Anna, woman 69 years)

Moreover, the participants’ experiences highlight that accessing and participating in rehabilitation should be as low-threshold as possible. Based on the experiences of the study participants, access to rehabilitation may be hindered by various types of barriers: structural barriers such as long travel distances or transportation challenges; functional barriers such as difficulties with the registration process; and informational barriers, such as uncertainty about the content of the rehabilitation.

*But since everything requires using a phone*,* you must register for them weeks in advance and at a specific time*,* so I often don’t do it*,* and I don’t go.* (Lisa, woman 83 years)

*I haven’t been able to go to the chair exercise*,* no*,* no. I don’t really know what it’s like there*,* because I don’t know what chair exercises are.* (Bertha, woman 93 years)

### Main theme 2: need for individually tailored rehabilitation

Based on the study participants’ responses, positive encounters and feelings of being heard may be interpreted as foundation for individualisation. As the following quotation shows, a person with dementia needs to feel being heard, and individual psychological and health-related characteristics should be considered, as these can influence enthusiasm and motivation towards rehabilitation.

*I was rehabilitated when I was in the hospital after the fall*,* and there was a rehabilitation therapist*,* a physical therapist who was a bit like someone from the army in the sense that he really commanded me and tried to force me to do those movements. I remember that I was pretty much against it because it hurt so much.* (Anna, woman 69 years)

The participants’ experiences reflect that when a person is genuinely encountered and heard, it becomes possible to consider their individual characteristics, which may relate to motivation and capacity to engage in rehabilitation. Individual characteristics include, for example, personal resources, overall health condition, readiness to engage in rehabilitation and physical health issues, such as pain, poor vision, or dizziness. These individual characteristics are not always immediately apparent. Therefore, through pleasant encounters, it becomes possible to identify and consider these individual characteristics.

*Yeah*,* they did try to rehabilitate me*,* but I guess I gave the impression that I’m not really interested. And I’m not interested*,* because rehabilitation causes pain.* (Anna, woman 69 years)

*Of course*,* it depends on one’s own condition how much he/she can participate.* (Maria, woman 86 years)

The content of rehabilitation should be meaningful and adaptable to the individual’s functional capacity and physical health condition. Rehabilitation becomes more meaningful when it reflects the individual’s personal needs and interests. Such experiences can foster satisfaction with oneself and strengthen the sense of self-efficacy, which can lead to increased motivation and commitment to rehabilitation. Rehabilitation content seems important in motivating individuals and supporting their engagement and offering alternative activities to replace those that can no longer be performed due to changes in functional capacity. The meaningful rehabilitation can involve, for example, therapist-guided exercises, linking exercises to daily activities, walking or group exercise.

*And is the rehabilitation offered suitable for their condition? … When I went to group exercise*,* I faced a situation where the exercises were done in the middle of the floor. I fell down all the time so now I go to chair exercises.* (Kate, woman 86 years)

*I have that routine*,* let’s say*,* so walking that route feels quite natural*,* fortunately. I even feel a sense of pleasure within myself when I’m outside of these four walls.* (Emily, woman 90 years)

### Main theme 3: need for social support

The participants’ experiences revealed a need for distributed social support encompassing both health care professionals and their loved ones, such as family members, friends, and peers. Social support was perceived as vital for enhancing motivation and commitment to rehabilitation, enabling the continuity of rehabilitation, and supporting mental well-being. The participants’ experiences indicated that they understood the importance of rehabilitation but carrying it out was perceived as challenging without present guidance, support and encouragement of another person.

Social support from their loved ones seems important in compensating for the study participants’ cognitive challenges, for example by reminding them to carry out exercises and remain physically active. Having another person present also provides a sense of safety and engaging in activities together with another person or as a part of a group seems motivating and supportive for participation.

*It’s going to happen when there’s a friend involved. For me at least*,* that’s how it is; I’m so lazy. But when I have a friend to go with*,* then I go.* (Emily, woman 90 years)

The participants described that they valued professional guidance in rehabilitation, including clear explanations and justifications for activities and their benefits. They reported experiencing such support as motivating and helping them to engage in rehabilitation and take responsibility for their rehabilitation.

*I’ve also received very good instructions*,* and under the nurse’s guidance*,* I stood up whenever possible to rehabilitate myself.* (Simone, woman 92 years)

However, the participants’ experiences also reflect that health care professionals’ attitudes may have an impact on an individual’s opportunities to be physically active and engage in rehabilitation. The following quotation is a good example of the need for encouragement and that sometimes professionals’ attitudes can even be a barrier to rehabilitative activities.

*At home*,* I do walk on my own*,* but here (in day care)*,* they won’t let me; they don’t allow it. They say I’ll fall and hurt myself. That really bothers me.* (William, man 84 years)

Although rehabilitation was largely perceived as physical rehabilitation, the connection between the mind and body and the impact of mental well-being was recognised and expressed as desirable by the study participants. They linked mental well-being to meaningful encounters with others and ongoing social support seems meaningful for mental well-being and for the continuity of rehabilitation. At times, the presence of another person appeared even more important than the rehabilitation itself.

*When the nurses have a moment to spare*,* even if there isn’t anything particularly meaningful to discuss*,* it branches out into a nice moment. These encounters are quite significant for mental rehabilitation—it would certainly be better if we could focus more on the body instead of talking*,* but it affects the body as well.* (Maria, woman 86 years)

## Discussion

The aim of this qualitative study was to understand the perceived rehabilitation needs of older people with dementia. The results highlight the importance of well-coordinated, individually tailored rehabilitation and social support as facilitators of successful rehabilitation. These rehabilitation needs of older people with dementia should be met to facilitate successful rehabilitation. Previous studies have identified challenges in implementing rehabilitation for people with dementia [39, 40], requiring a deeper understanding of how to integrate rehabilitation into their daily lives successfully.

The participants of this study perceived rehabilitation mostly as physical activity and physical rehabilitation. Therefore, our findings focus on physical rehabilitation, although the results can be most likely generalised more broadly to rehabilitation. Moreover, people with dementia seem to consider rehabilitation mostly as physical rehabilitation and maintaining functional ability [25]. This may, on the one hand, reflect the importance of physical functioning among older individuals, and on the other hand, a narrow self-perception of rehabilitation among people living with dementia.

It has also been previously reported that younger persons with dementia tend to have broader views on rehabilitation goals, including supporting independence and quality of life [25]. Reasons for the various perceptions remain unclear, but they may be related, for instance, to age. Overall, rehabilitation is a complex and broad concept that can be difficult to define—not only for people with dementia, but also for those around them. Enhancing understanding of rehabilitation among individuals with dementia, their close ones, and health care professionals may help in identifying rehabilitation needs and improving access to appropriate services.

The study participants’ experiences reflect that identifying the rehabilitation needs and access to rehabilitation appear fragmented and hindered by multiple barriers, such as structural factors, functional barriers and informational barriers. In addition, the study participants described that rehabilitation is offered selectively rather than systematically. The results are similar to previous studies; people with dementia report seeking services and facing barriers to rehabilitation access on multiple levels [25, 43]. Dementia can lead to apathy and impair a person’s initiative and motivation [49]. Therefore, as the results of this study also suggest, dementia increases the need for social support in identifying the need for rehabilitation, accessing rehabilitation services, and sustaining motivation and engagement. As dementia brings challenges in recognising one’s own situation [50], the role of professionals and family members in identifying needs and facilitating access to rehabilitation becomes essential. Moreover, reduced initiative and motivation further emphasise the need for social support and individually tailored, participatory rehabilitation that considers the specific needs of the person with dementia.

Meeting the rehabilitation needs of people with dementia requires the involvement of professionals from different fields of rehabilitation [17, 35]. In our study some participants found it meaningful to integrate rehabilitation into daily routines. Therefore, the expertise of an occupational therapist may be beneficial in linking rehabilitation to everyday life. Working in comprehensive teams facilitates the delivery of individualised rehabilitation, supports coordinated care, and promotes continuity across the rehabilitation process [17].

Our findings suggest that, in addition to professionals, support from the person’s loved ones may also be necessary to ensure the successful implementation of rehabilitation. Lindelöf et al. (2023) have also noted that implementing rehabilitation for people with dementia requires support from society, relatives and professionals [17]. Especially for persons with dementia, social support is crucial to enable engagement in and continuation with rehabilitative activities [16, 17, 44]. In our study, people with dementia experienced a lack of motivation and companionship as barriers to participation and continuing rehabilitation, especially when considering self-directed activities. Therefore, social support appears vital to facilitate successful rehabilitation for this group of older people. Our findings reinforce the need for interdisciplinary team-based rehabilitation that also involves the loved ones of the person with dementia.

On the other hand, involving informal caregivers and interdisciplinary healthcare professionals may also be challenging due to balancing diverse expectations [17]. Therefore, future research should aim to identify effective strategies for the implementation of distributed social support in rehabilitation. This entails, for instance, finding ways to provide the desired support while considering the perspectives and preferences of different stakeholders, including people with dementia, their caregivers, and healthcare professionals.

Previous studies demonstrate undeniable benefits of individually tailored rehabilitation among older people with dementia [9, 44, 51]. Our findings also highlight the importance of individually tailored rehabilitation. In our study, people with dementia tied individuality with the importance of being heard, through which it becomes possible to consider a person’s overall situation and personal interest. It seems that through individualisation, it can be possible to implement adaptable and meaningful rehabilitation, which can enhance commitment and motivation towards rehabilitation and increase the self-efficacy of people with dementia. Similarly, Sondell et al. (2021), in a study exploring the experiences of community-dwelling older people with dementia participating in a person-centred, multidimensional, interdisciplinary rehabilitation program, found that being seen and acknowledged by staff and participating in a group strengthened participants’ motivation and sense of self-efficacy [44].

Personalised goal setting in rehabilitation is an important part of individuality [26], and home-dwelling people with dementia consider their individual needs and goals [25] and the individualisation of exercises as important [44]. In previous studies, goal setting has been identified as feasible and as a central component of rehabilitation for people with dementia [26, 27]. Our findings strengthen the view that people with dementia can express their own rehabilitation needs.

Previously, Ries (2018) introduced a well-structured and practical framework of rehabilitation for physiotherapists to facilitate therapeutic success [42]. The key elements of this framework are establishing personal relationships, using intentional verbal and nonverbal communication, understanding and optimising motor learning, and creating a safe and purposeful environment. Our results can possibly complement this framework by considering factors beyond the therapy session, starting with recognising the needs and referral to rehabilitation and emphasising individuality and distributed social support as key elements for successful rehabilitation. Overall, the results of our study can be used by healthcare professionals working with people with dementia, especially since rehabilitation should be seen not only as daily physical rehabilitation but also as a background philosophy of dementia care.

### Strengths and limitations

Some limitations of the study need to be considered. When interviewing people with dementia, it is important to consider the possible influence of their cognitive changes on their responses. Especially for people with dementia, defining rehabilitation can be challenging due to its complexity and broadness. However, we sought to take this into account in designing and implementing the study. To strengthen trustworthiness during data collection, we considered the participants’ cognitive decline by opting for a relatively small group size for focus group interviews (3–4 participants) [52], paying attention to simple interview questions [53], conducting the interviews in a calm environment and warming up the interview session with an informal coffee setting [54]. This helped to create a good, trusting connection with the participants. In addition, a short informal discussion was held after each focus group session to maintain a good atmosphere after the discussion. Based on the richness and depth of the interview data, group dynamics seemed to have a stimulating effect on the conversation, and it appeared that participants were able to share their experiences and thoughts freely and in depth. During the analysis phase, reflexive practices were applied, and interpretations were discussed within the research group to enhance intersubjective validity.

The use of a healthcare professional as a gatekeeper in participant recruitment can be seen as either a strength or a limitation. On the one hand, the gatekeeper’s involvement likely facilitated a group discussion with functional group dynamics. On the other hand, relying on a gatekeeper’s decision may have caused selection bias. It is important to acknowledge that a potential limitation of this study is that participants were recruited through a specific service provider. It is possible that the providers’ organisational practices can shape the participants’ experiences of access to rehabilitation. Consequently, the transferability of the findings to other contexts should be considered with caution.

## Conclusions

The number of people living with dementia will increase rapidly in the coming decades due to an ageing population. As most dementia cases are diagnosed early, promoting the health and independence of people living with these diseases is crucial. Our study revealed important aspects from the participants’ own perspective, which need to be considered to provide successful rehabilitation. Our results are in line with the core idea of the disability movement, *“nothing about us without us”*, which has also been highlighted by several Alzheimer’s associations. Our results highlight the significance of clarifying rehabilitation pathways and ensuring the availability of rehabilitation services based on subjective rehabilitation needs and preferences expressed by older people with dementia. Additionally, awareness of rehabilitation opportunities should be increased among people with dementia and their families, as well as formal and informal caregivers. The social support from family caregivers, friends and peers and healthcare professionals seems vital for the successful implementation and engagement of rehabilitation. Individually tailored rehabilitation should include being encountered and heard, consideration of a person’s overall situation and capacities, as well as identification of goals, needs, challenges, and life histories. Through this approach, rehabilitation can become meaningful, motivating and engaging for people living with dementia.

## Supplementary Information

Additional file 1: Interview guide of the focus group discussions. This file provides the interview questions that guided the focus group discussions

## Data Availability

The datasets used and analysed during the current study are available from the corresponding author on reasonable request.

## Abbreviations

ADL: Activities of daily living
WHO: World Health Organization

## References

[1] Laver K, Dyer S, Whitehead C, Clemson L, Crotty M. Interventions to delay functional decline in people with dementia: a systematic review of systematic reviews. BMJ Open. 2016;27(4):e010767. 10.1136/bmjopen-2015-010767. 10.1136/bmjopen-2015-010767 PMC4854009 27121704

[2] Gauthier S, Webster C, Servaes S, Morais J, Rosa-Neto P. World alzheimer report 2022 – Life after diagnosis: navigating treatment, care and support. London, England: Alzheimer’s Disease International; 2022. https://www.alzint.org/resource/world-alzheimer-report-2022/.

[3] World Health Organization. Dementia: a public health priority. Geneva: World Health Organization; 2012. https://www.alzint.org/resource/dementia-a-public-health-priority/.

[4] Ascher-Svanum H, Chen YF, Hake A, Kahle-Wrobleski K, Schuster D, Kendall D, et al. Cognitive and functional decline in patients with mild alzheimer dementia with or without comorbid diabetes. Clin Ther. 2015;1(6):1195–205. 10.1016/j.clinthera.2015.01.00. 10.1016/j.clinthera.2015.01.002 25676448

[5] Solomon A, Dobranici L, Kåreholt I, Tudose C, Lăzărescu M. Comorbidity and the rate of cognitive decline in patients with alzheimer dementia. Int J Geriatr Psychiatry. 2011;26(12):1244–51. 10.1002/gps.2670. 21500282 10.1002/gps.2670

[6] Abreu W, Tolson D, Jackson GA, Staines H, Costa N. The relationship between frailty, functional dependence, and healthcare needs among community-dwelling people with moderate to severe dementia. Health Soc Care Commun. 2019;27(3):642–53. 10.1111/hsc.12678. 10.1111/hsc.12678 30402986

[7] Doroszkiewicz H. How the cognitive status of older people affects their care dependency level and needs: A Cross-Sectional study. Int J Environ Res Public Health. 2022;19(16):10257. 10.3390/ijerph191610257. 36011890 10.3390/ijerph191610257 PMC9408506

[8] Spijker A, Vernooij-Dassen M, Vasse E, Adang E, Wollersheim H, Grol R, et al. Effectiveness of nonpharmacological interventions in delaying the institutionalization of patients with dementia: a meta-analysis. J Am Geriatr Soc. 2008;56(6):1116–28. 10.1111/j.1532-5415.2008.01705.x. 18410323 10.1111/j.1532-5415.2008.01705.x

[9] Pitkälä KH, Pöysti MM, Laakkonen ML, Tilvis RS, Savikko N, Kautiainen H, et al. Effects of the Finnish alzheimer disease exercise trial (FINALEX): a randomized controlled trial. JAMA Intern Med. 2013;27(10):894–901. 10.1001/jamainternmed.2013.359. 10.1001/jamainternmed.2013.359 23589097

[10] Liu L, Dong H, Jin X, Brooke-Wavell KBW. Tackling dementia: A systematic review of interventions based on physical activity. J Geriatric Phys Ther. 2022;45(4). 10.1519/JPT.0000000000000332. 10.1519/JPT.0000000000000332 34570041

[11] Lam FM, Huang MZ, Liao LR, Chung RC, Kwok TC, Pang MY. Physical exercise improves strength, balance, mobility, and endurance in people with cognitive impairment and dementia: a systematic review. J Physiother. 2018;64(1):4–15. 10.1016/j.jphys.2017.12.001. 29289581 10.1016/j.jphys.2017.12.001

[12] World Health Organization. Global action plan on the public health response to dementia 2017–2025. Geneva: World Health Organization; 2017. https://iris.who.int/handle/10665/259615.

[13] Clare L. Rehabilitation for people living with dementia: A practical framework of positive support. PLoS Med. 2017;7(3):e1002245. 10.1371/journal.pmed.1002245. 10.1371/journal.pmed.1002245 PMC5340348 28267744

[14] Poulos CJ, Bayer A, Beaupre L, Clare L, Poulos RG, Wang RH, et al. A comprehensive approach to reablement in dementia. Alzheimers Dement (N Y). 2017;3(3):450–8. 10.1016/j.trci.2017.06.005. 29067351 10.1016/j.trci.2017.06.005 PMC5654482

[15] World Health Organization. Package of interventions for rehabilitation: module 3: neurological conditions. Package of interventions for rehabilitation for dementia. 2023. https://www.who.int/publications/i/item/9789240071131

[16] Maki Y, Sakurai T, Okochi J, Yamaguchi H, Toba K. Rehabilitation to live better with dementia. Geriatr Gerontol Int. 2018;18(11):1529–36. 10.1111/ggi.13517. 30318671 10.1111/ggi.13517

[17] Lindelöf N, Nilsson I, Littbrand H, Gustafson Y, Olofsson B, Fjellman-Wiklund A. A focus groups study of staff team experiences of providing interdisciplinary rehabilitation for people with dementia and their caregivers—a co-creative journey. BMC Geriatr. 2023;18(1):572. 10.1186/s12877-023-04269-3. 10.1186/s12877-023-04269-3 PMC10507912 37723442

[18] Gupta A, Prakash NB, Sannyasi G. Rehabilitation in dementia. Indian J Psychol Med. 2021 Sept;43(5 Suppl):S37–47. 10.1177/02537176211033316 PMC8543618 34732953

[19] Clare L, Kudlicka A, Oyebode JR, Jones RW, Bayer A, Leroi I, et al. Goal-oriented cognitive rehabilitation for early-stage alzheimer’s and related dementias: the GREAT RCT. Health Technol Assess. 2019;23(10):1–242. 30879470 10.3310/hta23100 PMC6441850

[20] Dörr F, Holle D, Morouj B, Obermüller D, Sommer S, Wübbeler M, et al. Speech-language therapy and occupational therapy for patients with mild cognitive impairment and dementia: a retrospective cohort study using German health claims data. BMC Health Serv Res. 2025;25(1):1026. 40764599 10.1186/s12913-025-13149-y PMC12326587

[21] Bennett S, Laver K, Voigt-Radloff S, Letts L, Clemson L, Graff M, et al. Occupational therapy for people with dementia and their family carers provided at home: a systematic review and meta-analysis. BMJ Open. 2019;9(11):e026308. 10.1136/bmjopen-2018-026308. 31719067 10.1136/bmjopen-2018-026308 PMC6858232

[22] World Health Organization. Rehabilitation in health systems. Geneva: World Health Organization. 2017. https://iris.who.int/handle/10665/254506

[23] Gutenbrunner C, Meyer T, Melvin J, Stucki G. Towards a conceptual description of physical and rehabilitation medicine. J Rehabil Med. 2011;43(9):760–4. 10.2340/16501977-0866. 21874211 10.2340/16501977-0866

[24] Thuesen J, Ravn MB, Petersen KS. Towards person-centred rehabilitation in dementia – a narrative synthesis. Disabil Rehabil. 2021;28(18):2673–9. 10.1080/09638288.2019.1709910. 10.1080/09638288.2019.1709910 31906726

[25] Laver KE, Crotty M, Low LF, Clemson L, Whitehead C, McLoughlin J, et al. Rehabilitation for people with dementia: a multi-method study examining knowledge and attitudes. BMC Geriat. 2020;9(1):531. 10.1186/s12877-020-01940-x. 10.1186/s12877-020-01940-x PMC7727118 33297973

[26] Dutzi I, Schwenk M, Kirchner M, Bauer JM, Hauer K. What would you like to achieve? Goal-Setting in patients with dementia in geriatric rehabilitation. BMC Geriat. 2019;22(191):280. 10.1186/s12877-019-1296-7. 10.1186/s12877-019-1296-7 PMC6805571 31640595

[27] Jogie P, Rahja M, van den Berg M, Cations M, Brown S, Laver K. Goal setting for people with mild cognitive impairment or dementia in rehabilitation: A scoping review. Aust Occup Ther J. 2021;68(6):563–92. 10.1111/1440-1630.12758. 34346077 10.1111/1440-1630.12758

[28] Feng F, Xu H, Sun Y, Zhang X, Li N, Sun X, et al. Exercise for prevention of falls and fall-related injuries in neurodegenerative diseases and aging-related risk conditions: a meta-analysis. Front Endocrinol. 2023;14:14:1187325. 10.3389/fendo.2023.1187325. 10.3389/fendo.2023.1187325 PMC10393124 37534209

[29] Law CK, Lam FM, Chung RC, Pang MY. Physical exercise attenuates cognitive decline and reduces behavioural problems in people with mild cognitive impairment and dementia: a systematic review. J Physiother. 2020;1(1):9–18. 10.1016/j.jphys.2019.11.014. 10.1016/j.jphys.2019.11.014 31843427

[30] Hasselgren L, Conradsson M, Lampinen J, Toots A, Olofsson B, Nilsson I, et al. Feasibility of a person-centred multidimensional interdisciplinary rehabilitation programme in community-dwelling people with dementia: a randomised controlled pilot trial. BMC Geriat. 2024;28(241):794. 10.1186/s12877-024-05372-9. 10.1186/s12877-024-05372-9 PMC11439292 39342131

[31] Begde A, Jain M, Hogervorst E, Wilcockson T. Does physical exercise improve the capacity for independent living in people with dementia or mild cognitive impairment: an overview of systematic reviews and meta-analyses. Aging Ment Health. 2022;2(12):2317–27. 10.1080/13607863.2021.2019192. 10.1080/13607863.2021.2019192 PMC9662184 34951548

[32] Thoma-Lürken T, Bleijlevens MHC, Lexis MAS, de Witte LP, Hamers JPH. Facilitating aging in place: A qualitative study of practical problems preventing people with dementia from living at home. Geriatr Nurs. 2018;39(1):29–38. 10.1016/j.gerinurse.2017.05.003. 28624128 10.1016/j.gerinurse.2017.05.003

[33] Martyr A, Nelis SM, Quinn C, Rusted JM, Morris RG, Clare L. The relationship between perceived functional difficulties and the ability to live well with mild-to‐moderate dementia: findings from the IDEAL programme. Int J Geriatr Psychiatry. 2019;34(8):1251–61. 10.1002/gps.5128. 31034650 10.1002/gps.5128 PMC6767698

[34] Clare L, Wu YT, Jones IR, Victor CR, Nelis SM, Martyr A, et al. A comprehensive model of factors associated with subjective perceptions of living well with dementia. Alzheimer Dis Assoc Disord. 2019;33(1):36–41. 10.1097/WAD.0000000000000286. 30802227 10.1097/WAD.0000000000000286 PMC6416010

[35] Cations M, Laver KE, Crotty M, Cameron ID. Rehabilitation in dementia care. Age Ageing. 2018;1(2):171–4. 10.1093/ageing/afx173. 10.1093/ageing/afx173 29194465

[36] Cations M, May N, Crotty M, Low LF, Clemson L, Whitehead C et al. Health Professional Perspectives on Rehabilitation for People With Dementia. Gerontologist. 2020; 2;60(3):503–12. 10.1093/geront/gnz007 10.1093/geront/gnz007 30759218

[37] Isbel ST, Jamieson MI. Views from health professionals on accessing rehabilitation for people with dementia following a hip fracture. Dement (London). 2017;16(8):1020–31. 10.1177/1471301216631141. 10.1177/1471301216631141 26843421

[38] Mitchell R, Harvey L, Brodaty H, Draper B, Close J. Hip fracture and the influence of dementia on health outcomes and access to hospital-based rehabilitation for older individuals. Disabil Rehabil. 2016;38(23):2286–95. 10.3109/09638288.2015.1123306. 26765956 10.3109/09638288.2015.1123306

[39] Scerri A, Innes A, Scerri C. Healthcare professionals’ perceived challenges and solutions when providing rehabilitation to persons living with dementia-A scoping review. J Clin Nurs. 2023;32(17–18):5493–513. 10.1111/jocn.16635. 36710398 10.1111/jocn.16635

[40] Quick SM, Snowdon DA, Lawler K, McGinley JL, Soh SE, Callisaya ML. Physical therapist and physical therapist student Knowledge, Confidence, Attitudes, and beliefs about providing care for people with dementia: A Mixed-Methods systematic review. Phys Ther. 2022;5(5):pzac010. 10.1093/ptj/pzac010. 10.1093/ptj/pzac010 PMC9155993 35157773

[41] Goodwin VA, Allan LM. Mrs Smith has no rehab potential’: does rehabilitation have a role in the management of people with dementia? Age Ageing. 2019;1(1):5–7. 10.1093/ageing/afy152. 10.1093/ageing/afy152 30247498

[42] Ries JD. Rehabilitation for individuals with dementia: facilitating success. Curr Geri Rep. 2018;1(1):59–70. 10.1186/s40945-022-00134-5.

[43] Layton N, Devanny C, Hill K, Swaffer K, Russell G, Low LF, et al. The right to rehabilitation for people with dementia: A codesign approach to barriers and solutions. Health Expect. 2024;27(5):e70036. 10.1111/hex.70036. 39318228 10.1111/hex.70036 PMC11422665

[44] Sondell A, Lampinen J, Conradsson M, Littbrand H, Englund U, Nilsson I et al. Experiences of community-dwelling older people with dementia participating in a person-centred multidimensional interdisciplinary rehabilitation program. BMC Geriatr. 2021; 2;21(1):341. 10.1186/s12877-021-02282-y 10.1186/s12877-021-02282-y PMC8173830 34078266

[45] Leplege A, Gzil F, Cammelli M, Lefeve C, Pachoud B, Ville I. Person-centredness: conceptual and historical perspectives. Disabil Rehabil. 2007;15(20–21):1555–65. 10.1080/09638280701618661. 10.1080/09638280701618661 17922326

[46] Finlay L. Phenomenology for therapists: researching the lived world. Hoboken, N.J: J. Wiley; 2011.

[47] Braun V, Clarke V. Using thematic analysis in psychology. Qualitative Res Psychol. 2006;1(2):77–101. 10.1191/1478088706qp063oa.

[48] Braun V, Clarke V. Thematic Analysis. A practical guide. Sage; 2021.

[49] Cheng YT, Xin GK, Wang YL, Tan FY, Yuan L, Zhang Y et al. The current status of apathy in patients with dementia and its factors: A systematic review. Geriat Nurs 2024 July 1;58:290–7. 10.1016/j.gerinurse.2024.05.022 10.1016/j.gerinurse.2024.05.022 38848610

[50] Wilson RS, Systma J, Barnes LL, Boyle PA. Anosognosia in dementia. Curr Neurol Neurosci Rep. 2016;16:77. 10.1007/s11910-016-0684-z. 27438597 10.1007/s11910-016-0684-z

[51] van der Wardt V, Hancox J, Gondek D, Logan P, Nair R das, Pollock K, et al. Adherence support strategies for exercise interventions in people with mild cognitive impairment and dementia: A systematic review. Prev Med Rep. 2017;18(7):38–45. 10.1016/j.pmedr.2017.05.007. 10.1016/j.pmedr.2017.05.007 PMC5447393 28593121

[52] Krueger RA. Focus groups: A practical guide for applied research. SAGE; 2014.

[53] Cridland EK, Phillipson L, Brennan-Horley C, Swaffer K. Reflections and recommendations for conducting In-Depth interviews with people with dementia. Qual Health Res. 2016;1(13):1774–86. 10.1177/1049732316637065. 10.1177/1049732316637065 27055496

[54] James M. Wilkinson Heather. Make it Easy on Yourself! 2004;3(2):117–125. 10.1177/14713012040423322

