# Supplementary material for: Perceived rehabilitation needs of older people with dementia: A qualitative interview study
Source: BMC Geriatr. 2025 Nov 7;25:863. doi: 10.1186/s12877-025-06570-9 (PMC12595687; doi:10.1186/s12877-025-06570-9)
Supplement: Supplementary file 1 — Additional file 1: Interview guide of the focus group discussions. This file provides the interview questions that guided the focus group discussions [file 12877_2025_6570_MOESM1_ESM.pdf]

Additional file 1

The interview guide of the focus group discussions.

What does the word “Rehabilitation” bring to your mind?

Can you tell me about your experiences of rehabilitation in your life?

Please, describe your own wishes related to rehabilitation.

How are your wishes related to rehabilitation visible in your everyday life?

What helps you to feel well in your everyday life?

What kind of support do you wish for your well-being?

How do you experience the possible support you have received for your well-being?

Can you tell me about your experiences with rehabilitation services?

What kind of challenges, if any, have you encountered in relation to rehabilitation?
